# Plumericin Protects against Experimental Inflammatory Bowel Disease by Restoring Intestinal Barrier Function and Reducing Apoptosis

**DOI:** 10.3390/biomedicines9010067

**Published:** 2021-01-12

**Authors:** Shara Francesca Rapa, Rosanna Di Paola, Marika Cordaro, Rosalba Siracusa, Ramona D’Amico, Roberta Fusco, Giuseppina Autore, Salvatore Cuzzocrea, Hermann Stuppner, Stefania Marzocco

**Affiliations:** 1 Department of Pharmacy, University of Salerno, Via Giovanni Paolo II 132, 84084 Fisciano, Italy; srapa@unisa.it (S.F.R.); autore@unisa.it (G.A.); 2 Department of Chemical, Biological, Pharmaceutical and Environmental Sciences, University of Messina, 98168 Messina, Italy; dipaolar@unime.it (R.D.P.); rsiracusa@unime.it (R.S.); rdamico@unime.it (R.D.); rfusco@unime.it (R.F.); salvator@unime.it (S.C.); 3 Department of Biomedical, Dental, Morphological and Functional Imaging Sciences, University of Messina, Via Consolare Valeria, 98125 Messina, Italy; cordarom@unime.it; 4 Institute of Pharmacy/Pharmacognosy, Center for Molecular Biosciences Innsbruck (CMBI), University of Innsbruck, Innrain 80/82, 6020 Innsbruck, Austria; Hermann.Stuppner@uibk.ac.at

**Keywords:** plumericin, inflammatory bowel disease, intestinal epithelial cells, experimental colitis, intestinal barrier, apoptosis

## Abstract

Intestinal epithelial barrier impairment plays a key pathogenic role in inflammatory bowel diseases (IBDs). In particular, together with oxidative stress, intestinal epithelial barrier alteration is considered as upstream event in ulcerative colitis (UC). In order to identify new products of natural origin with a potential activity for UC treatment, this study evaluated the effects of plumericin, a spirolactone iridoid, present as one of the main bioactive components in the bark of *Himatanthus sucuuba* (Woodson). Plumericin was evaluated for its ability to improve barrier function and to reduce apoptotic parameters during inflammation, both in intestinal epithelial cells (IEC-6), and in an animal experimental model of 2, 4, 6-dinitrobenzene sulfonic acid (DNBS)-induced colitis. Our results indicated that plumericin increased the expression of adhesion molecules, enhanced IEC-6 cells actin cytoskeleton rearrangement, and promoted their motility. Moreover, plumericin reduced apoptotic parameters in IEC-6. These results were confirmed in vivo. Plumericin reduced the activity of myeloperoxidase, inhibited the expression of ICAM-1, P-selectin, and the formation of PAR, and reduced apoptosis parameters in mice colitis induced by DNBS. These results support a pharmacological potential of plumericin in the treatment of UC, due to its ability to improve the structural integrity of the intestinal epithelium and its barrier function.

## 1. Introduction

Inflammatory bowel diseases (IBD), mainly including ulcerative colitis (UC) and Crohn’s disease (CD), are complex chronic inflammatory conditions that can be debilitating and sometimes lead to life-threatening complications [1,2]. The IBD pathogenesis is complex and multifactorial (e.g., environmental, infectious, immunological, psychological, and genetic) [3].

UC is characterized by continuous colonic inflammation with loss of normal vascular pattern, erythema, erosions, granularity, bleeding, friability, and ulcerations, with distinct demarcation between inflamed and non-inflamed bowel [4]. The classical histological changes in UC include decreased crypt density, crypt architectural distortion, irregular mucosal surface, and heavy diffuse transmucosal inflammation and impairments in intestinal epithelial barrier function [4]. 

The intestinal epithelial barrier plays a pivotal role in maintaining intestinal homeostasis. The epithelial layer, in conjunction with the mucosal layer and specialized cells, forms a well-equipped, intricately regulated, and stringent barrier with continuous scrutiny by immune cells to create an immune-silent environment [5]. The intestinal epithelial barrier disruption allows the entry of pro-inflammatory molecules, such as pathogens, toxins, and antigens, from the luminal environment into the mucosal tissues and circulatory system [6]. Thus, it contributes to the development of intestinal inflammation, as in IBD and in particular in UC [7]. In this scenario, intestinal epithelial cells (IECs) play a pivotal role responding to microbial stimuli to reinforce the barrier function and participating in the coordination of an appropriate immune and inflammatory response [8]. 

The single layer of IECs, forming the intestinal barrier, are sealed by the intracellular junctions that modulate intestinal permeability by regulating the paracellular transport of water and ions. Intracellular junctions also represent the first line of defense against the entry of noxious agents that, once transported via the paracellular route, can initiate local inflammation, and, once in the circulation, they can also promote systemic tissue inflammation and damage [9,10,11]. Junctions’ barrier disruption and increased paracellular permeability, followed by permeation of luminal pro-inflammatory molecules, can induce activation of the mucosal immune system, contributing to sustained inflammation, tissue damage during colitis, and other related complications [12,13,14]. The sustained inflammation induces IECs apoptosis suggesting that TNF-mediated pathways play key roles in inducing this programmed cell death [15].

Standard UC therapies including anti-inflammatory, immunosuppressive, and biological treatments are widely used, but due to the low remission rate and the severe side effects of these therapies, there is increasing interest in new therapeutic interventions in IBD patients [7,16,17].

Plant-derived natural products significantly contributed to drug discovery in the past and still provide an effective source for lead structure identification [18]. In South America, preparations of the stem bark and latex of the Amazonian tree *Himatanthus sucuuba* (Spruce ex Müll.Arg.) Woodson (Apocynaceae) have been traditionally used as anti-inflammatory, antitumor, analgesic, and antiulcer agents [19]. Plumericin, a major bioactive constituent of *H. sucuuba*, is a spirolactone iridoid and has been shown to exhibit antiparasitic [20], antimicrobial [21], and antifungal [22] activities. Plumericin has been shown to be a potent NF-κB pathway inhibitor [23], to have antiproliferative properties in the vasculature [24], and to reduce TNF-α-induced senescence of endothelial cells [25]. More recently this natural compound has been reported to reduce inflammation and oxidative stress during intestinal inflammation [26]. Thus, in order to further characterize the plumericin potential in IBD, in this study, we evaluated its effect on intestinal barrier and apoptosis in a model of intestinal inflammation both in vitro and in vivo.

## 2. Experimental Section

### 2.1. Reagents

Unless otherwise specified, all reagents and compounds were purchased from Sigma Chemicals Company (Sigma, Milan, Italy).

### 2.2. Plant Material

Plumericin was isolated from the bark of *H. sucuuba*. Detailed description of the phytochemical work including the isolation and identification of plumericin and other compounds from *H. sucuuba* has previously been provided elsewhere [27]. Details about the storage, stability, and purity of the plumericin have previously been described [26].

### 2.3. In Vitro Studies

#### 2.3.1. Cell Culture

Intestinal epithelial cells, IEC-6 (CRL-1592), was purchased from the American Type Culture Collection (ATCC). IEC-6 cell line, deriving from rat small intestinal crypt, was cultured using Dulbecco’s modified Eagle’s medium (4 g/L glucose) supplemented with 10% (*v*/*v*) fetal bovine serum, 2 mM l-glutamine, 1.5 g/L NaHCO_3_, and 0.1 U/mL bovine insulin. Cells were grown at 37 °C in a humidified atmosphere of 5% CO_2_/95% air, and the viability was monitored using phase contrast microscopy and trypan blue staining. Cells were used at the 16–19th passage.

#### 2.3.2. Establishment of An In Vitro IEC-6 Cell Model of Inflammatory Injury

In order to establish a cellular model of inflammatory damage, IEC-6 cells were plated and, after 24 h of adhesion, at right confluence, were treated with plumericin (0.5–2 µM) for 1 h and then co-exposed to plumericin and two pro-inflammatory stimuli, such as lipopolysaccharides from *E. coli* (LPS; 10 μg/mL) plus interferon-γ (IFN; 10 U/mL) for different times, based on the mediator to evaluate [28].

#### 2.3.3. Measurement of Claudin-1, Occludin, E-Cadherin, Bax, Bcl-2, Bcl-xL and Caspase-3 Expression by Cytofluorimetry

IEC-6 cells were plated into 96-well plates (2.0 × 10^3^ cells/well) and treated with plumericin (0.5–2 µM), as previously indicated, for 24 h. For this analysis, IEC-6 cells were then collected and washed with phosphate buffered saline (PBS). Fixing solution was added to cells for 20 min and then IEC-6 cells were incubated in fix perm solution for a further 30 min. Anti-Claudin-1 (Thermofisher Scientific, Waltham, MA, USA), anti-Occludin (Thermofisher Scientific, Waltham, MA, USA), anti-E-cadherin (Cell Signaling Technology, Dellaertweg, The Netherlands), anti-Bax (Santa Cruz Biotechnologies, Dallas, TX, USA), anti-Bcl-2 (Santa Cruz Biotechnologies, Dallas, TX, USA), anti-Bcl-xL (Thermofisher Scientific, Waltham, MA, USA), or anti-caspase 3 (Thermofisher Scientific, Waltham, MA, USA) antibodies were then added for 1 h. The secondary FITC-conjugated antibody, in fixing solution, was added to IEC-6 cells and cell fluorescence was then evaluated by a fluorescence-activated cell sorter (FACSscan; Becton Dickinson, Milan, Italy) and analyzed by Cell Quest software (version 4; Becton Dickinson, Milan, Italy), as formerly reported [29].

#### 2.3.4. Immunofluorescence Assay for Cytoskeleton Analysis by Confocal Microscopy

To evaluate plumericin effects on cellular cytoskeleton, IEC-6 cells were seeded on coverslips in 12 well plate (2.0 × 10^5^ cells/well) and treated with plumericin (1 μM) alone or in combination with LPS + IFN, for 24 h. Cells were fixed with 4% paraformaldehyde in PBS for 15 min and permeabilized with 0.1% saponin in PBS for 15 min. Slides were then incubated with FITC-conjugated anti-F-actin (Phalloidin-FITC, Sigma, Milan, Italy) at the concentration of 1 mg/mL in PBS for 30 min. The slides were then washed three times with PBS and DAPI was used for counterstaining of nuclei. Coverslips were finally mounted in mounting medium and fluorescent images were taken under the Laser Confocal Microscope (Leica TCS SP5, Wetzlar, Germany), as previously described [30].

#### 2.3.5. Wound Healing Assay 

In order to evaluate IEC-6 cellular migration a wound-healing assay was performed, as previously reported [31]. IEC-6 cells (1.0 × 10^5^ cells/well, 24-well plates) were allowed to adhere for 24 h. After 100% of confluence, a mechanical scratch was induced at the center of the IEC-6 monolayer by gently scraping cells with a sterile plastic p10 pipette tip. Cells were then washed with PBS and treated with plumericin (0.5–2 μM) alone or in combination with LPS + IFN for 24 h. After the scratch, IEC-6 cells were placed in a humidified and equilibrated (5% *v*/*v* CO_2_) incubation chamber of an Integrated Live Cell Workstation Leica AF-6000 LX at 37 °C for 24 h. A 10× phase contrast objective was used in order to record cell movements, with a frequency of acquisition of 10 min. To determine the migration rate of individual cells, we considered the distances covered from the initial time to the selected time points (bar of distance tool, Leica AF software, 2.3.5 build 5379, Leica, Wetzlar, Germany). For each scratch, at least three different positions were registered and, to measure the migration distances, for each position, at least 10 different cells were randomly selected. GraphPad Prism 5 software (GraphPad, San Diego, CA, USA) was used to perform the statistical analyses.

#### 2.3.6. Analysis of Apoptosis

The anti-apoptotic activity of plumericin was analyzed by evaluating the percentage of hypodiploid DNA, using propidium iodide (PI) staining by flow cytometry [32]. Briefly, IEC-6 cells (3.5 × 10^5^) were grown in 24-well plates and allowed to adhere. Thereafter cells were exposed to plumericin (0.5–2 μM) for 1 h and then co-exposed to plumericin and LPS + IFN for further 24 h. Following treatment, culture medium was removed, cells washed once with PBS, and then resuspended in 500 µL of a solution containing 0.1% (*w*/*v*) sodium citrate and 50 µg/mL PI. Culture medium and PBS were centrifuged, and cell pellets were pooled with cell suspension to retain both dead and living cells for analysis. After incubation at 4 °C for 30 min in the dark, cell nuclei were analyzed with a Becton Dickinson FACScan flow cytometer (FACSscan; Becton Dickinson, Milan, Italy) using the CellQuest program (version 4; Becton Dickinson, Milan, Italy).

### 2.4. In Vivo Studies

#### 2.4.1. Animals 

Male CD1 mice (20–25 g, Harlan Nossan, Milan, Italy) were housed in a controlled environment, maintained on a 12-h light/dark cycle, and received a standard rodent chow and water, available ad libitum. The University of Messina Review Board for Animal Care (OPBA) approved the study (650/2017-PR). Animal care was in conformity with current legislation of the EU for the protection of animals used for scientific purposes (Directive 2010/63/EU).

#### 2.4.2. Induction of Experimental Colitis

Colitis was induced by intrarectal administration of 2,4,6-dinitrobenzene sulfonic acid (DNBS, 4 mg per mouse) [33]. In preliminary experiments, this dose of DNBS was found to induce reproducible colitis without mortality. Mice were anesthetized by Enflurane. Subsequently, DNBS (4 mg in 100 μL of 50% ethanol/50% saline, *v*/*v*) was injected into the rectum through a catheter inserted 4.5 cm proximally to the anus. Vehicle alone (100 μL of 50% ethanol/50% saline) was administered in control experiments (control). Thereafter, the animals were kept for 15 min in a Trendelenburg position. After colitis induction, the animals were observed for 4 days. On day 4, the animals were weighed and anaesthetized with chloral hydrate, and the abdomen was opened by a midline incision. The colon was removed, freed from surrounding tissues, opened along the antimesenteric border, washed, weighed, and processed for histologic and biochemical studies. Plumericin (3 mg/kg; 0.2% DMSO) was intraperitoneally (i.p.) administered.

#### 2.4.3. Experimental Groups

Animals were casually divided into several groups (*n* = 10 for each group):

- Control + vehicle group: Vehicle solution was given by i.p. administration each day for 4 days;
- Control + plumericin (3 mg/kg) group: Plumericin was administered i.p. each day for 4 days (data not shown);
- DNBS + vehicle group: DNBS was injected to the mice as described, subsequently, vehicle solution was administered i.p. each day for 4 days; the first dose was injected 1 h after the injection of DNBS;
- DNBS + plumericin (3 mg/kg) group: DNBS was injected to the mice as described, subsequently, plumericin (3 mg/kg) was given i.p. each day for 4 days; the first dose was injected 1 h after the administration of DNBS.

#### 2.4.4. Immunohistochemical Localization of ICAM-1, P-Selectin and PAR

At 4 days after DNBS administration, colon tissues were fixed in 10% (*w*/*v*) PBS-buffered formaldehyde and 7 μm sections were prepared from paraffin embedded tissues. After deparaffinization, endogenous peroxidase was quenched with 0.3% (*v*/*v*) H_2_O_2_ in 60% (*v*/*v*) methanol for 30 min. The sections were permeabilized with 0.1% (*w*/*v*) Triton X-100 in PBS for 20 min. Non-specific adsorption was minimized by incubating the section in 2% (*v*/*v*) normal goat serum in PBS for 20 min. Endogenous biotin or avidin binding sites were blocked by sequential incubation for 15 min with biotin and avidin (Vector Laboratories, Burlingame, CA, USA), respectively. Colon tissue sections were incubated overnight with anti-ICAM-1 (Santa Cruz Biotechnology, Dallas, TX, USA), anti-P-selectin 1 (Santa Cruz Biotechnology), or anti-PARP (Santa Cruz Biotechnology). Colon sections were rinsed with PBS and incubated with peroxidase-conjugated bovine anti-mouse immunoglobulin G (IgG) secondary antibody or peroxidase-conjugated goat anti-rabbit IgG (Jackson Immuno Research, West Grove, PA, USA). Specific labelling was detected with a biotin-conjugated goat anti-rabbit IgG or biotin-conjugated goat anti-mouse IgG and avidin-biotin peroxidase complex (Vector Laboratories, Burlingame, CA, USA). To verify the binding specificity for all antibodies, control slices were incubated with only primary antibody or secondary antibody. In these controls, no positive staining was detected. Immunohistochemical images were collected using a Zeiss microscope and Axio Vision software. For graphic display of densitometric analyses, the intensity of positive staining (brown staining) was measured by computer-assisted color image analysis (Leica QWin V3, London, UK). The percentage area of immunoreactivity (determined by the number of positive pixels) was expressed as percent of total tissue area (red staining). Replicates for each experimental condition and histochemical staining were obtained from each mouse in all experimental groups. All immunohistochemical analyses were carried out by 2 observers blinded to the treatment [34].

#### 2.4.5. Myeloperoxidase Assay 

Neutrophil infiltration in the colon was examined by measuring tissue myeloperoxidase (MPO) activity using a spectrophotometric assay with tetramethylbenzidine as substrate, according to a previously published method [35]. After DNBS injection, colon tissues were collected and weighed. Each piece of tissue was homogenized in a solution containing 0.5% hexa-decyl-trimethyl-ammonium bromide dissolved in 10 mM potassium phosphate buffer pH 7 and centrifuged for 30 min at 20,000× *g* at 4 °C. An aliquot of the supernatant was then allowed to react with a solution of 1.6 mM tetramethylbenzidine and 0.1 mM hydrogen peroxide (H_2_O_2_). The rate of change in absorbance was measured spectrophotometrically at 650 nm. MPO activity was described as the quantity of enzyme degrading 1 μmol of peroxide per min at 37 °C and was expressed in U/g wet tissue.

#### 2.4.6. Bax and Bcl-2 Determination by Western Blot Analysis from Colon Tissue

Tissue samples from the terminal colon were suspended in extraction Buffer A containing 0.2 mM PMSF, 0.15 μM pepstatin A, 20 μM leupeptin, 1 mM sodium orthovanadate, homogenized at the highest setting for 2 min, and centrifuged at 1000× *g* for 10 min at 4 °C. Supernatants represented the cytosolic fraction. The pellets, containing enriched nuclei, were re-suspended in Buffer B containing 1% Triton X-100, 150 mM NaCl, 10 mM Tris-HCl pH 7.4, 1 mM EGTA, 1 mM EDTA, 0.2 mM PMSF, 20 μM leupeptin, and 0.2 mM sodium orthovanadate. After centrifugation for 30 min at 15,000× *g* at 4 °C, the supernatants containing the nuclear protein were stored at −80 °C for further analysis. The following primary antibodies were used: anti-Bax (Santa Cruz Biotechnology), anti-Bcl-2 (Santa Cruz Biotechnology) at 4 °C overnight in 1 × PBS, 5% (*w*/*v*), non-fat dried milk, and 0.1% Tween-20. After, membranes were incubated with peroxidase-conjugated bovine anti-mouse IgG secondary antibody or peroxidase-conjugated goat anti-rabbit IgG (Jackson ImmunoResearch, West Grove, PA, USA) for 1 h at room temperature. To ascertain that blots were loaded with equal amounts of protein lysates, they were also incubated in the presence of the antibody against β-actin (Santa Cruz Biotechnology). Signals were detected with enhanced chemiluminescence detection system reagent according to manufacturer’s instructions (Super Signal West Pico Chemiluminescent Substrate, Pierce, Altrincham, UK). The relative expression of the protein bands was quantified by densitometry with Bio-Rad ChemiDoc XRS + software and standardized to β-actin levels. Images of blot signals (8-bit/600-dpi resolution) were imported to analysis software (Image Quant TL, v2003, Altrincham, UK), as previously reported [36].

### 2.5. Data Analysis and Statistical Evaluation

Data are reported as mean ± standard error of the mean (SEM) of at least three independent experiments. Each experiment was conducted in triplicate. For the in vivo studies, N represents the number of animals used. In the experiments involving histology or immunohistochemistry, the figures shown are demonstrative of at least three experiments. Statistical analyses were performed using the variance test. Bonferroni’s test was used to make multiple comparisons. A *p*-value of less than 0.05 was considered significant.

## 3. Results

### 3.1. Plumericin Increased Adhesion Molecules Expression in LPS + IFN-Stimulated IEC-6

**Figure 1:**
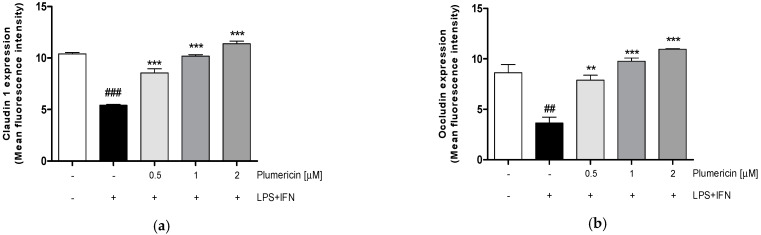

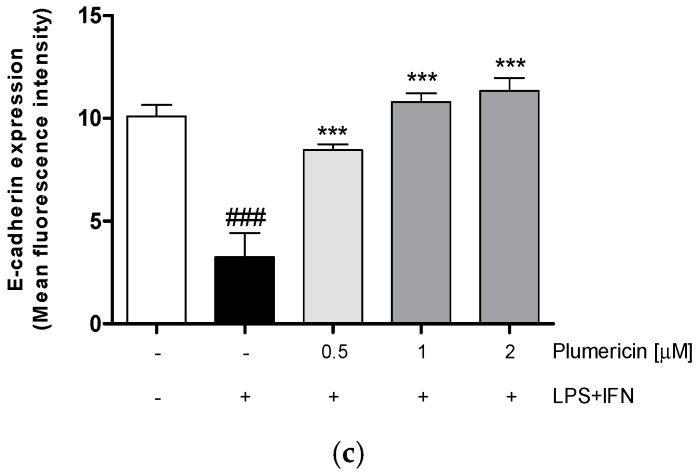
Effect of plumericin on adhesion molecules: Claudin 1 (**a**), Occludin (**b**), and E-cadherin (**c**) expression in LPS + IFN-stimulated IEC-6, evaluated by flow cytometry. Values are expressed as mean ± SEM of mean fluorescence intensity. ### and ## denote, respectively, *p* < 0.001 and *p* < 0.01 vs. untreated cells; *** and ** indicate, respectively, *p* < 0.001 and *p* < 0.01 vs. LPS + IFN.

Mucosal inflammation as observed in colitis compromises the epithelial barrier, resulting in the exposure of lamina propria to luminal antigens and microbes that further contribute to the inflammatory response and barrier defects [37]. Adhesion proteins play a key role in maintaining the integrity of the intestinal epithelial barrier. However, several studies have reported differential effects of inflammatory mediators on adhesion proteins during IBD [38]. In order to assess plumericin’s ability to modulate the expression of adhesion molecules, such as Claudin-1, Occludin, and E-cadherin, in inflammatory conditions, we analyzed their expression by flow cytometry. Our results indicated that plumericin (0.5–2 µM) significantly increased Claudin-1 (*p* < 0.001 vs. LPS + IFN; Figure 1a), Occludin (*p* < 0.001 vs. LPS + IFN; Figure 1b), and E-cadherin (*p* < 0.001 vs. LPS + IFN; Figure 1c) expression, in LPS + IFN stimulated IEC-6.

### 3.2. Plumericin Enhanced IEC-6 Cells Actin Cytoskeleton Rearrangement Induced by LPS + IFN

**Figure 2:**
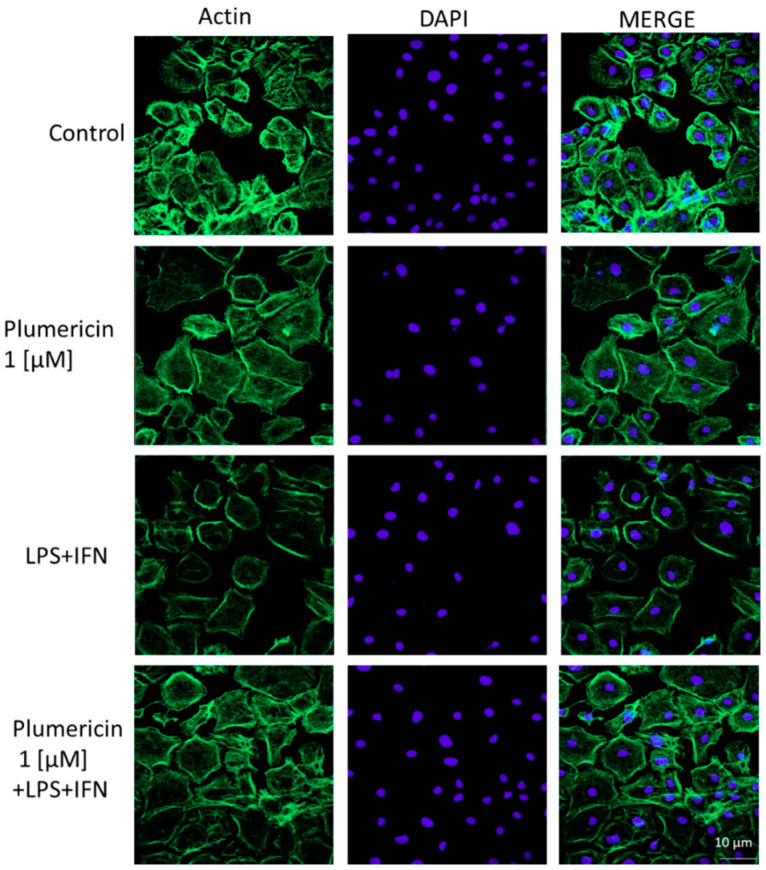
Effect of plumericin (1 µM) on the actin stress fibers assembly in IEC-6 cells. Immunofluorescence analysis was performed using immunofluorescence confocal microscopy. Scale bar = 10 µm. Green fluorescence indicated the localization of actin stress fibers and blue fluorescence indicated the localization of nuclei (DAPI). The MERGE indicates an overlap of the two fluorescences.

During the restitution of IECs, extensive reorganization of the actin cytoskeleton is necessary. For this reason, confocal microscopy was used to examine the effect of plumericin (1 µM) in normal and in inflammatory conditions on the assembly of actin stress fibres, that play a crucial role in the remodeling, stretching, and migration of epithelial cells. Untreated cells showed an intact actin cytoskeleton and its fibers traversing the cytosol. Addition of LPS + IFN caused a weak reorganization of actin filaments characterized by their redistribution to the cell subcortical compartment and subsequent cell. Moreover, in the presence of LPS + IFN, plumericin produced a reversion of this effect (Figure 2).

### 3.3. Plumericin Promoted IEC-6 Motility

**Figure 3:**
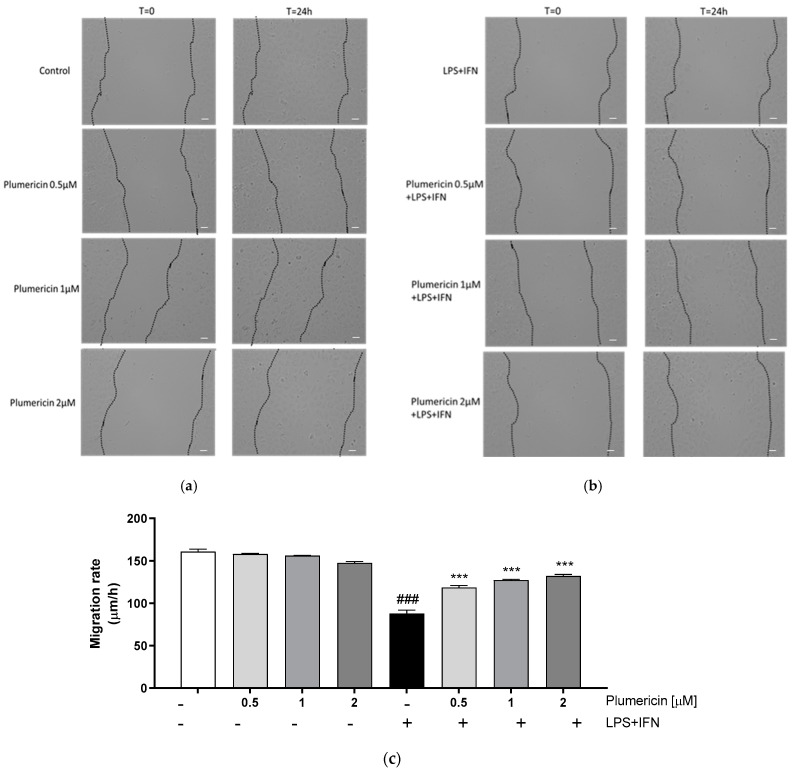
Effect of plumericin alone and in presence of LPS + IFN on cellular migration, evaluated by wound-healing assay. Pictures representing the wound repair, induced by mechanical scratch in IEC-6, from plumericin treatment alone (**a**) and in combination with LPS + IFN (**b**). Bar = 150 µm. (**c**) representing the quantitative analysis expressed as IEC-6 migration rate after 24 h. Values are expressed as migration rate (µm/h). ### indicates *p* < 0.001 vs. untreated cells; *** indicates *p* < 0.001 vs. LPS + IFN.

During colitis, intestinal wound healing is dependent on the precise balance of migration, proliferation, and differentiation of the epithelial cells adjacent to the wounded area. In order to assess the effect of the plumericin on the reconstitution process at the intestinal level, we carried out a wound-healing assay, to evaluate cellular migration, on treated IEC-6 monolayers. Our results indicated that plumericin (0.5–2 µM) alone did not significantly alter IEC-6 migration speed compared to untreated cells (*p* < 0.001 vs. control; Figure 3a,c). However, in inflammatory conditions, plumericin demonstrated significant improvement in the speed of IEC-6 migration, compared to LPS + IFN-treated cells (*p* < 0.001 vs. LPS + IFN; Figure 3b,c).

### 3.4. Plumericin Reduced LPS + IFN-Induced Apoptosis

**Figure 4:**
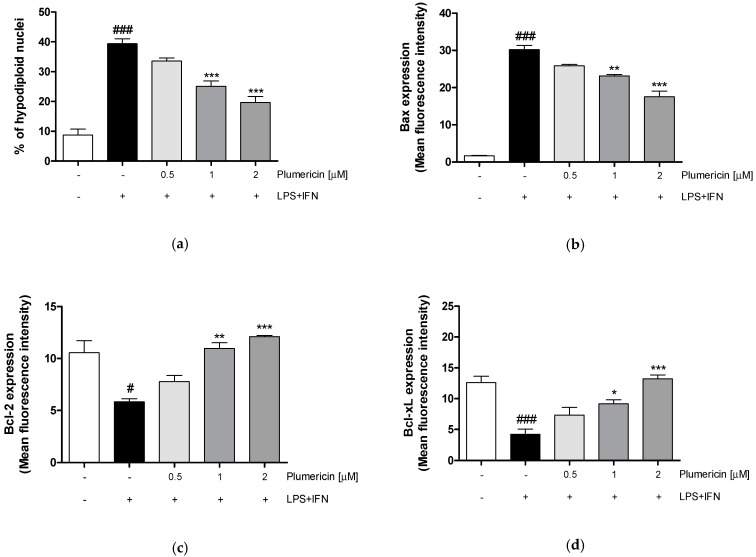

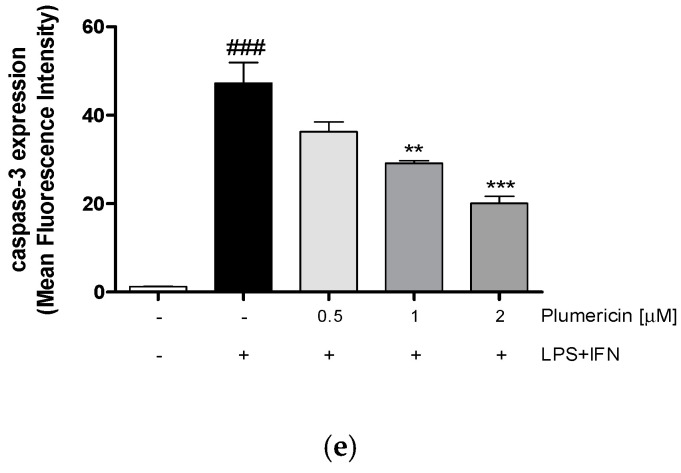
Apoptosis detection by propidium iodide (PI) staining of hypodiploid nuclei (**a**), and effect on Bax (**b**), Bcl-2 (**c**), Bcl-xL (**d**), and Caspase-3 (**e**), after IEC-6 incubation with plumericin. Data are expressed as mean ± S.E.M. of % of hypodiploid nuclei or as mean of fluorescence intensity. ### and # denote, respectively, *p* < 0.001 and *p* < 0.05 vs. untreated cells; ***, **, and * denote, respectively, *p* < 0.001, *p* < 0.01, and *p* < 0.05 vs. LPS + IFN.

In order to investigate the mechanisms underlying the gastroprotective effect of plumericin, we evaluated apoptosis, by cytofluorimetric analysis of PI stained hypodiploid nuclei. Our results indicated that plumericin (0.5–2 µM) significantly reduced LPS + IFN-induced apoptosis in IEC-6 cells, at higher concentrations tested (Figure 4a; *p* < 0.001 vs. LPS + IFN). 

To further investigate the anti-apoptotic ability of plumericin, we evaluated the expression of Bax, a pro-apoptotic protein, and Bcl-2 and Bcl-xL, two anti-apoptotic proteins, by cytofluorimetric analysis. Our results indicated that plumericin (0.5–2 μM) significantly reduced Bax expression (*p* < 0.01 vs. LPS + IFN; Figure 4b) and increased Bcl-2 and Bcl-xL expression in LPS + IFN-stimulated IEC-6 (*p* < 0.01 vs. LPS + IFN; Figure 4c,d), at higher concentrations tested. 

Plumericin’s anti-apoptotic ability was further demonstrated by evaluating caspase-3 expression, with flow cytometry. Our results have shown that plumericin significantly reduced the expression of caspase-3, compared to LPS + IFN-treated cells (*p* < 0.01 vs. LPS + IFN; Figure 4e)

### 3.5. Effect of Plumericin on ICAM-1 and P-Selectin Expression in DNBS-Induced Colitis

**Figure 5:**
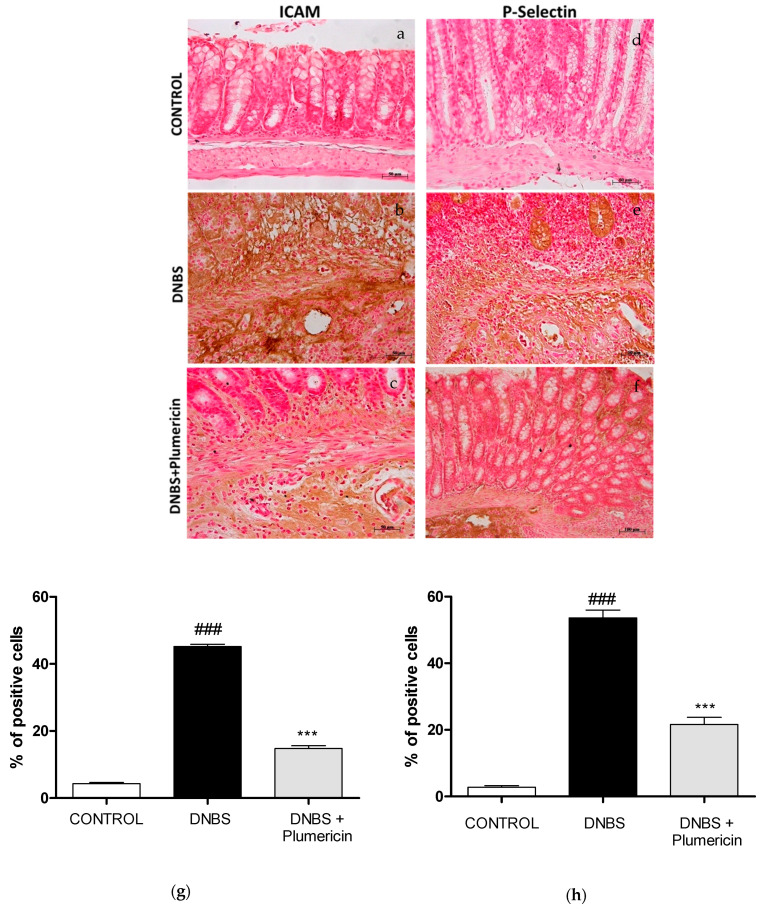
Effect of plumericin on ICAM-1 (**a**–**c**) and P-selectin (**d**–**f**) expression. (**g**,**h**) represent, respectively, densitometric analysis expressed as percentage of total tissue area. Photographs are representative of all animals in each group and data are means ± S.E.M. of 10 mice for each group. ### denotes *p* < 0.001 vs. control; *** denotes *p* < 0.001 vs. DNBS.

In this study, we also evaluated the intestinal expression of ICAM-1 and P-selectin that contribute to cell recruitment during colon inflammation. Positive staining for ICAM-1 (Figure 5b) and for P-selectin (Figure 5e) was substantially increased in the vessels of the lamina propria and submucosa as well as in epithelial cells of injured colon and in infiltrated inflammatory cells in damaged tissues from DNBS-injected mice. Treatment with plumericin (3 mg/kg; i.p.) reduced the staining for ICAM-1 (Figure 5c) and for P-selectin (Figure 5f) in the colon tissues collected from DNBS-injected mice. No positive staining for ICAM-1 and for P-selectin was observed in the colon tissues collected from control mice (Figure 5a,d, respectively), as you can see from the densitometric analyses, respectively, in Figure 5g,h.

### 3.6. Plumericin Treatment Reduced MPO Activity in DNBS-Induced Colitis

**Figure 6:**
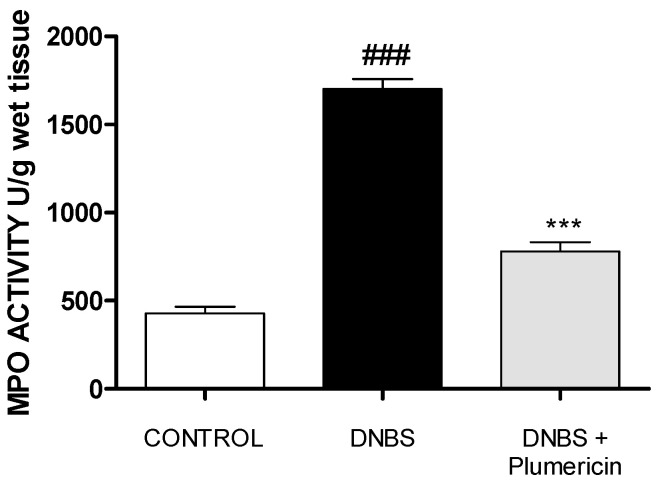
Effect of plumericin on myeloperoxidase (MPO) activity. Data are expressed as the mean ± S.E.M. of 10 mice for each group. ### denotes *p* < 0.001 vs. control group; *** denotes *p* < 0.001 vs. DNBS.

DNBS intrarectally administration was characterized by an augmentation in MPO activity, an indicator of neutrophil accumulating in the colon. This was consistent with light microscopic observations showing the colon of vehicle-treated DNBS mice to contain a large number of neutrophils. In contrast, plumericin (3 mg/kg, i.p.) significantly reduced the degree of polymorphonuclear cell infiltration (determined as reduction in MPO activity) in inflamed colon (*p* < 0.001 vs. DNBS; Figure 6).

### 3.7. Effect of Plumericin Treatment on PAR Formation in Colitis Induced by DNBS

**Figure 7:**
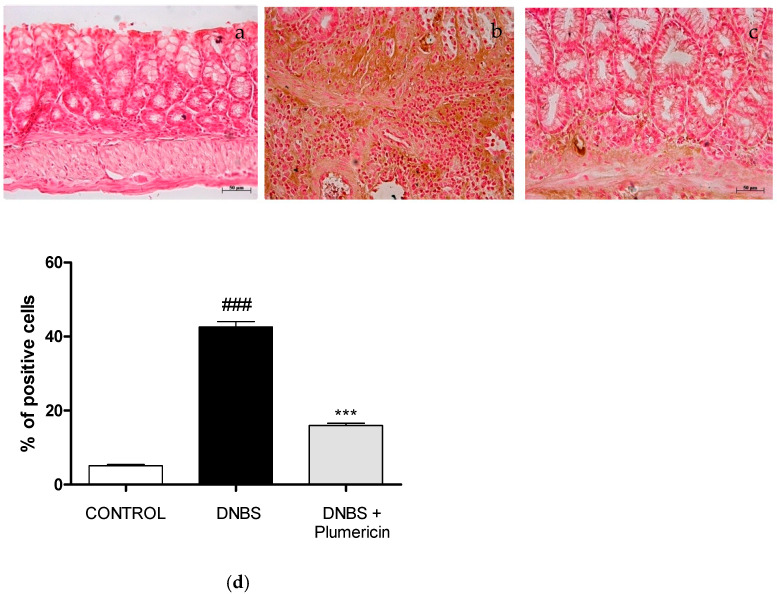
Effects of plumericin treatment on PAR formation (**a**–**c**) in colitis induced by DNBS. These data are also visible in graph of the percentage of total tissue area (**d**). Photographs are representative of all animals in each group. Data are expressed as the mean ± S.E.M. of 10 mice for each group. ### denotes *p* < 0.001 vs. control; *** denotes *p* < 0.001 vs. DNBS.

Immunohistochemistry for PAR, as an indicator of in vivo PARP activation, revealed positive staining localized in the nuclei of inflammatory cells in colon tissues from DNBS-injected mice (Figure 7b). Plumericin (3 mg/kg, i.p.) significantly reduced the extent of PAR immunoreactivity in the colon (Figure 7c), four days after DNBS administration. No positive staining for PAR was found in the colon tissues from control mice (Figure 7a), as you can see from the densitometric analyses (Figure 7d).

### 3.8. Effect of Plumericin on Apoptotic Damage in DNBS-Induced Colitis

**Figure 8:**
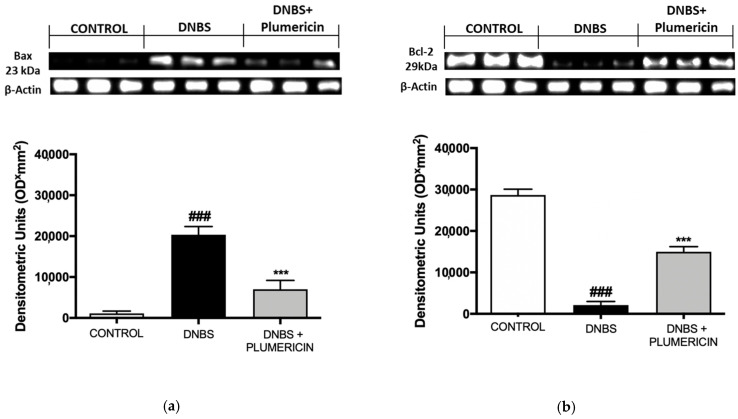
Bax (**a**) and Bcl-2 (**b**) expression was measured by Western blot. Densitometric analysis of protein bands was normalized to the level of β-actin. Data are representative of at least three independent experiments and are expressed as mean ± S.E.M. from 10 animals for each experimental group. A representative blot is shown and densitometric analysis is reported. ### denotes *p* < 0.001 vs. control; *** denotes *p* < 0.001 vs. DNBS.

To test whether colon damage was also associated with apoptosis, four days after DNBS, the appearance of proteic effectors of canonical mitochondrial apoptosis such as pro-apoptotic (Bax) protein and anti-apoptotic (Bcl-2) protein, was investigated by Western blot analysis. The balance of Bax levels was appreciably increased in the colon from mice subjected to DNBS. On the contrary, plumericin treatment (3 mg/kg; i.p.) prevented DNBS-induced Bax expression (*p* < 0.001 vs. DNBS; Figure 8a). Moreover, in the control groups, a basal level of Bcl-2 was detected. In DNBS-injected mice, Bcl-2 expression was reduced, but plumericin administration showed an increase in Bcl-2 expression (*p* < 0.001 vs. DNBS; Figure 8b).

## 4. Discussion

UC is a chronic progressive condition and impose significant multidimensional burdens on patients and health care systems. At the turn of the 21st century, IBDs as UC have become a global disease with accelerating incidence in newly industrialized countries whose societies have become more westernized [1,2,3,4]. A great interest is focused on new therapies that may be beneficial in treating these chronic diseases and the use of plant products for treating UC has increased, as many of them are known to act on different targets of the pathology process such as inflammation, oxidative stress, and intestinal barrier function [39,40]. In this study, we focused the attention on the effect of plumericin, an iridoid spironolactone isolated from *H. sucuuba*, on the management of the intestinal barrier impairment and apoptosis during intestinal inflammation. 

A “leaky gut” may be an initial event in the pathogenesis of IBDs and it may also perpetuate chronic mucosal inflammation in UC flaring up uncontrollable inflammatory signal cascades and the altered expression of intracellular junction proteins is observed in patients with UC [41]. Epithelial tight junctions (TJs) and adherens junctions (ADJ) forms a selectively permeable seal between adjacent epithelial cells and define the limit between the apical and basolateral membrane domain. Their impairment allows the passage of pro-inflammatory molecules and can induce a mucosal immune system activation finally resulting in a sustained inflammation and tissue damage [5,42]. Accordingly, in our experimental model, we observe an impaired claudin-1, occludin, and E-cadherin expression in LPS + INF treated IEC-6. Plumericin treatment was able to avoid this impairment, reducing the decrease of these junction proteins expression in treated IEC-6 respect to the pro-inflammatory stimulus. 

The actin cytoskeleton is a master regulator of the assembly and remodeling of epithelial junctions and establishment of tissue barriers. The interaction of intracellular junction with the actin cytoskeleton is vital to the maintenance of junction structure and allow the barrier integrity regulation by cytoskeleton [43]. During inflammation, it is known that a reorganization of the apical junctions mediate epithelial barrier dysfunction and the actin cytoskeleton plays a pivotal role in regulating junctional integrity and remodeling under physiological and pathological states [44]. IEC-6 cells, when confluent, produce monolayers that resemble normal small intestinal cells and show an organized actin cytoskeleton [45,46]. In our experiments, we found that plumericin ameliorated the marked reduction of actin stress fibers and the corresponding increase of the cortical actin density observed in LPS + IFN-treated IEC-6. Recent studies suggest that balanced actin filament turnover protects epithelial barriers and attenuates tissue injury during mucosal inflammation in vivo [47]. 

A reorganization of the actin cytoskeleton is fundamental during IECs’ restitution. The healing process is initiated by migration of intestinal epithelial cells residing near the wounded area to the injury in order to fill up defects in the intestinal barrier. However, in various intestinal diseases, the intestinal healing is impaired, leading to persistent mucosal defects and potential consequences for the entire organism [48]. Thus, healing of the inflamed mucosa is considered a key step to achieve clinical remission in UC [41,49,50]. By means of a wound healing assay, we observed that plumericin improved cellular migration speed in IEC-6, thus increasing the restitution process. 

Apoptosis regulates the replacement rate of the epithelial cells, and an increase of epithelial cell apoptosis, as occurs in IBD and in colitis in particular, disrupts mucosal epithelial differentiation [51]. Our data indicate that plumericin inhibited the IEC-6 apoptosis. This effect was associated with a reduction in pro-apoptotic proteins, such as Bax, and with an increase in antiapoptotic factors, such as Bcl-2 and Bcl-xL. Moreover, in plumericin-treated IEC-6, a reduction in caspase-3 was also observed, thus further proving the anti-apoptotic effect of plumericin. 

In order to also confirm these effects observed in IECs in an in vivo model, we evaluated the effect of plumericin in a model of DNBS-induced colitis in mice. 

DNBS administration induced P-selectin expression on the endothelial vascular wall and up-regulated the surface expression of ICAM-1 on endothelial cells. In plumericin-treated mice, we observed a lower expression of P-selectin and an up-regulation of ICAM-1, without affecting constitutive levels of ICAM-1 on endothelial cells. The absence of an increased expression of the adhesion molecule in the colon tissue in plumericin-treated animals was associated with the reduction of leukocyte infiltration, as assessed by the specific granulocyte enzyme MPO, which is also an oxidative stress marker. 

ROS can cause DNA damage, leading to poly ADP ribose synthase activation and cell death [52]. Plumericin inhibited the positive staining for PAR compared to the DNBS-group. Epithelial cell damage in the inflamed colonic mucosa has been stated to involve the apoptotic process. In the DNBS group, we observed a significant increase in the pro-apoptotic protein Bax and a reduction in the antiapoptotic Bcl-2. Plumericin treatment induced both an inhibition of Bax expression and a parallel increase in Bcl-2, thus contributing to reducing the apoptotic process.

Plumericin shares its effects on these pathways with several other electrophilic compounds of high pharmaceutical and nutraceutical interest including dimethylfumarate and curcumin, which have also been reported to be effective on colitis [36,53,54]. This evidence further supports the pharmacological potential of plumericin as an adjuvant in IBDs.

## 5. Conclusions

The results of this study provide evidence, both in vitro and in vivo, that support the potential effect of plumericin on IBDs contributing to the maintenance of intestinal epithelial barrier and to reducing apoptosis. These findings together with the anti-inflammatory and antioxidant potential previously reported, strongly support the therapeutical potential of plumericin both in the acute phase of colitis, mainly acting on inflammation and oxidative stress, and also in the relapsing phase, where the restitution of intestinal barrier integrity seems to play a pivotal role.

## Data Availability

The data presented in this study are available on request from the corresponding author.
